# The plasminogen activator system: involvement in central nervous system inflammation and a potential site for therapeutic intervention

**DOI:** 10.1186/1742-2094-10-124

**Published:** 2013-10-11

**Authors:** Devorah Gur-Wahnon, Tehila Mizrachi, Florence-Yehudith Maaravi-Pinto, Athanasis Lourbopoulos, Nikolaos Grigoriadis, Abd -Al Roof Higazi, Talma Brenner

**Affiliations:** 1 Department of Neurology, Hadassah Medical Center, P.O. Box 12000, Jerusalem 91120, Israel; 2 Department of Neurology, Aristotle University Hospital, 1stilpe Kyriakidi str,, Thessaloniki GR 54636, Greece; 3 Department of Biochemistry, Hadassah Medical Center, P.O. Box 12000, Jerusalem 91120, Israel

## Abstract

**Background:**

Extracellular proteases such as plasminogen activators (PAs) and matrix metalloproteinases modulate cell-cell and cell-matrix interactions. Components of the PA/plasmin system have been shown to be increased in areas of inflammation, and have been suggested to play a role in inflammatory neurologic disorders such as epilepsy, stroke, brain trauma, Alzheimer's' disease and multiple sclerosis (MS). In the present study, we evaluated the involvement of the PA system in the animal model of MS, experimental autoimmune encephalomyelitis (EAE).

**Methods:**

EAE was induced by myelin oligodendrocyte glycoprotein (MOG) in mice deficient for the urokinase PA (uPA^−/−^), or the urokinase PA receptor (uPAR^−/−^). Mice were evaluated for EAE clinical signs and histopathologic parameters, and compared with wild-type (WT) EAE mice. Lymphocytes from the knockout (KO) and WT mice were analyzed for *ex vivo* restimulation, cytokine secretion, and antigen presentation. Finally, WT EAE mice were treated with PAI-1dp, an 18 amino acid peptide derived from the PA inhibitor protein (PAI-1).

**Results:**

EAE was aggravated in uPA^−/−^ and uPAR^−/−^ mice, and this was accompanied by more severe histopathologic features and microglial activation. By contrast, specific T- cell reactivity towards the encephalitogenic antigen MOG was markedly reduced in the KO animals, as shown by a marked reduction in proliferation and pro-inflammatory cytokine secretion in these mice. Antigen presentation was also reduced in all the KO animals, raising an immunologic paradox. When the mice were treated with PAI-1, a peptide derived from the PA system, a marked and significant improvement in EAE was seen. The clinical improvement was linked to reduced T-cell reactivity, further emphasizing the importance of the PA system in immunomodulation during neuroinflammation.

**Conclusions:**

Cumulatively, our results suggest a role for uPA and uPAR in EAE pathogenesis, as exacerbation of disease was seen in their absence. Furthermore, the successful amelioration of EAE by PAI-1 treatment suggests that the PA system can be considered a potential site for therapeutic intervention in the treatment of neuroimmune diseases.

## Introduction

Extracellular proteolysis represents a potent and irreversible mechanism of extracellular matrix remodeling [1]. It is involved in physiologic processes and in several pathologic conditions such as tumor invasion, inflammation, tissue repair, and excitoxicity [2,3]. Cumulative data have suggested that extracellular proteolysis plays a crucial role in the pathophysiology of neuronal cell death. Deleterious effects include disruption of the blood–brain barrier (BBB), amplification of inflammatory infiltrates, demyelination, and possibly disruption of cell-cell and cell-matrix interactions, which may trigger cell death. The positive effects of extracellular proteolysis include mediation of parenchymal and angiogenic recovery after brain injury [4].

The major extracellular proteolytic enzymes are the plasminogen activator (PA)/plasmin system and the matrix metalloproteinases (MMPs). PAs, specifically tissue PA (tPA) and urokinase-type PA (uPA), are serine proteases that cleave the zymogen plasminogen to generate the proteolytic enzyme plasmin. The PA/plasmin system has been implicated in fibrin removal, and in tissue remodeling and cell migration that occur during physiologic and pathologic processes [5], neurotoxicity, neuroprotection, and cerebral blood flow [5].

Studies on peripheral nerve injury have shown that fibrin deposition is a factor impeding axonal regeneration, whereas fibrin removal is associated with restoration of axonal function [6,7]. By contrast, activation of extracellular proteolysis is considered a pathogenic factor in demyelinating disorders such as multiple sclerosis (MS) and its animal model, experimental autoimmune encephalomyelitis (EAE). MS, an immune-mediated disease of the central nervous system (CNS), is characterized by chronic inflammatory processes. It involves the activation of CNS-immunocompetent cells and microglia, and extravasation of T cells and macrophages [8].

There is an ample body of literature regarding the involvement of PAs in neurologic disorders. Specifically, PA activity in MS is considered to play a major role in disturbance of the BBB and subsequent leukocyte migration, leading to inflammation, and causing myelin breakdown [9]. One of the earliest detectable signs of inflammatory demyelination is upregulation of urokinase type PA receptor (uPAR) and PA inhibitor (PAI-1). In the demyelinating MS lesion, tPA and uPA, forming a complex with PAI-1, are prominent in foamy macrophages, and are also a component of the MMP cascade. Significant parallel increases in components of the uPA system have been seen in MS tissue, with immunolocalization to macrophages in the active lesion, and mononuclear cells in the perivascular cuff [4,10]. Microglial cells isolated from the white matter of post-mortem MS tissue also have detectable uPAR [4,11]. Co-localization of uPAR with integrins on macrophages in the lesion further promotes adhesion to vitronectin, which in turn, leads to focal increases of PA activity [10]. During clinical EAE, the PA system was shown to be upregulated in the CNS as well [12]. Induction of tPA expression and of the PAI-1 transcript was detected in activated astrocytes in white matter. Inflammatory cells expressing uPAR also showed increased tPA and uPA activities in areas of inflammatory damage [12].

Activity of the PA system components is monitored closely and regulated by serine proteinase inhibitors, of which PAI-1 is most prominent. PAI-1 binds to active uPA and forms an uPA-PAI-1 complex, which is followed by the internalization and intracellular degradation of the complex [13].

In this study, we investigated the role of uPA and uPAR in EAE induced by myelin oligodendrocyte glycoprotein (MOG), by using uPA and uPAR knockout (KO) mice and comparing them with their wild-type (WT) counterparts. We found that the PA system affected the severity of EAE, with uPA^−/−^ and uPAR^−/−^ mice developing a more severe clinical course. The extent of inflammation, axonal loss, and axonal injury correlated with the more severe clinical outcome. Furthermore, pre-treating EAE mice with a PAI-1-derived peptide, (PAI-1-dp) [14] suppressed the development of EAE. Thus, our results support the importance of the PA components during neuroinflammation, and reveal potential sites for therapeutic intervention.

## Materials and methods

### Mice

C57BL/6 mice were purchased from Harlan Laboratories Ltd, Ein Kerem Breeding Farm, Jerusalem, Israel, and housed under specific pathogen-free conditions in the animal facility of the Hebrew University Medical School, Jerusalem, Israel, in accordance with National Institutes of Health (NIH) guidelines for the care and use of laboratory animals. KO mice deficient for uPA and uPAR against a C57BL/6 background were purchased (Jackson Immunoresearch Laboratories, West Grove, PA, USA), bred in the animal facility of the Hebrew University Medical School, and housed under specific pathogen-free conditions.

### EAE induction and clinical evaluation

EAE was induced in 8-week-old female C57BL/6 mice by subcutaneous injection into the left paralumbar region of 125 μg MOG _35–55_ peptide (synthesized by Sigma Laboratories, Israel) that had been emulsified in complete Freund’s adjuvant (CFA) containing 5 mg/ml heat-killed *Mycobacterium tuberculosis*. Immediately after this injection and, again 48 h later, the mice were inoculated intraperitoneally with 0.5 ml pertussis toxin (200 ng). An additional injection of MOG_35-55_ peptide in CFA was delivered 7 days later into the right paralumbar region.

All the animals were examined daily and evaluated for clinical signs of disease. The clinical status of the mice was graded as follows: 0, without clinical disease; 1, tail weakness; 2, hind limb weakness sufficient to impair righting; 3, single-limb plegia; 4, paraplegia with forelimb weakness; 5, quadriplegia; 6, death. According to the ethical requirements, mice that reached stage 4 were euthanaized.

### PAI-1-dp pre-treatment

An 18 amino acid peptide, Ac-RMAPEEIIMDRPFLYVVR-amide [14], derived from the PAI-1 protein (PAI-1-dp), was used for pre-treatment of mice induced with EAE (n = 16) and these were compared with control placebo-treated mice (n = 16). PAI-1-dp 0.5 mg/kg was injected intraperitoneal twice daily, starting 1 day preceding disease induction, and continued for 7 consecutive days. Mice were followed clinically as described above.

### Histopathology

Between days 17 and 25 post-induction, animals were transcardially perfused with paraformaldehyde in PBS (pH 7.2). The brains and spinal cords were removed, post-fixed in the same fixative, sectioned, and routinely processed for paraffin wax embedding and sectioning at 6 μm (coronal brain and longitudinal spinal-cord sections). The sections were then stained with a modified Bielschowsky silver impregnation combined with hematoxylin for simultaneous evaluation of axonal injury, axonal loss and infiltration, as previously described in detail [15,16].

Pathologic examination was performed under a light microscope (Axioplan-2; Zeiss), by two investigators blinded to the study groups. Photographs were captured with the aid of a digital camera (Nikon) attached to the microscope. For each animal, 10 randomly selected sections per tissue type (hemispheres, brain stem, cerebellum, and spinal cord), spaced at least 60 μm apart, were examined under high-power optical fields, using a prefrontal microscope grid as previously described [15]. Sections containing both gray and white matter were stained with hematoxylin and eosin. Inflammatory foci containing at least 20 perivascular mononuclear cells were assessed in each section. The number of perivascular and parenchymal infiltrates were counted, and the data were recomputed as infiltrates/mm^2^. Axonal injury (AI) was graded using the following scale 0, normal; 1+, a few scattered injured axons; 2+, focal mild to moderate AI; 3+, scattered mild to moderate AI or focal severe AI; 4+, scattered severe AI. Axonal loss (AL) was graded as: 0, normal; 1+, focal mild to moderate AL; 2+, scattered mild to moderate AL; 3+, focal severe AL and 4+, scattered severe AL.

### Microglial staining

Lectin-positive microglia/macrophages were detected in brain and spinal-cord sections using histochemical staining with biotin-labeled lectin (Sigma Aldrich, St. Louis, MO, USA) originating from *Lycopersicon esculent* (tomato), as previously described [15]. This molecule has been used previously for identifying microglia/macrophages and in revealing their morphology [17]. Briefly, sections were dewaxed, dried with ethanol, and treated with proteinase K (Clontec Laboratories, Mountain View, CA, USA) for 2.5 minutes at 37°C, followed by treatment with 0.3% H_2_O_2_ for 15 min and further incubation with 0.3% Triton-X in Tris-buffered saline containing an avidin-biotin blocking agent (Vector Laboratories, Burlingame, CA, USA). The sections were then incubated with the biotin-labeled lectin (3 μg/ml) for 2 hours at room temperature, and streptavidin was added to permit visualization with an appropriate chromogen (NovaRed; Vector Laboratories).

The lectin-positive microglia/macrophages were readily identified and morphologically distinguishable from blood vessels, which this molecule also stains [17,18]. The number of lectin-positive microglia/macrophages was counted, and the data were recomputed as lectin-positive cells/mm^2^.

### Lymphocyte proliferation assay

Pooled lymph-node cells (LNCs) were prepared from inguinal, axillary, and mesenteric lymph nodes or spleens derived from naive mice or from mice that had been subcutaneously inoculated 9 days earlier with MOG _35–55_ peptide in CFA. The *in vitro* response of the lymphocytes was assayed in triplicate wells of 96-well flat-bottom microtiter plates. A total of 2 × 10^5^ LNCs, suspended in 0.2 ml RPMI supplemented with 5% FCS, was added to each well with or without 100 μg/ml MOG _35–55_ peptide. At 48 h after seeding, 1 μCi ^3^[H]thymidine (Amersham Pharmacia Biotech, Amersham, Buckinghamshire, UK) was added to each well, and the plates were incubated for an additional 18 h. The plates were then harvested with a semi-automated harvester onto a glass fiber filter, and the radioactivity was determined by liquid scintillation assay.

### Antigen presentation assay

Antigen-presenting cells (APCs) were derived from spleens of WT, uPA^−/−^ or uPAR^−/−^ naive mice. Before co-culture with responder T cells, the splenocytes were irradiated at 3000 rad, so that the proliferation in the co-culture assays could be attributable solely to the responder T cells. The latter were obtained from MOG_35–55_-stimulated LNCs derived from MOG-immunized WT mice. The cells were maintained in MOG-containing medium for 4 days, after which the medium was replenished and supplemented with interleukin (IL)-2 (20 μg/ml) for an additional 5 days. For the antigen presentation assay, 50,000 responder T cells (per well) were co-cultured with 300,000 irradiated splenocytes derived from WT, uPA^−/−^ or uPAR^−/−^ naive mice, in the presence of MOG (50 μg/ml) for 72 h. The cells were cultured in RPMI 1640 containing 10% FCS supplemented with 5 × 10^−5^ mol/l 2-ME, 1 mmol/l sodium pyruvate, 1/100nonessential amino acids, 2mmole/L L-glutamine 2, and 100 U of penicillin/ml (Austria). Responder T-cell proliferation was assessed by [^3^H] thymidine incorporation.

### Statistical analysis

The histopathologic data were analyzed using SPSS software (version 18.0). The values of all the data are expressed as mean ± standard error (SE). Semi-quantitative data for the two groups (AI and AL) were analyzed using the Pearson χ^2^ test or Fisher’s exact test, where appropriate, and the data were displayed as bar graphs. The difference between the two groups was compared using the Mann–Whitney *U*-test or Student's *t*-test, as appropriate. The remaining data were analyzed using Student's t test and one-way ANOVA, according to Dunnett, and Fisher’s exact test. *P*<0.05 was considered significant.

## Results

### Aggravation of EAE in uPA^−/−^ and uPAR^−/−^ mice

**Figure 1:**
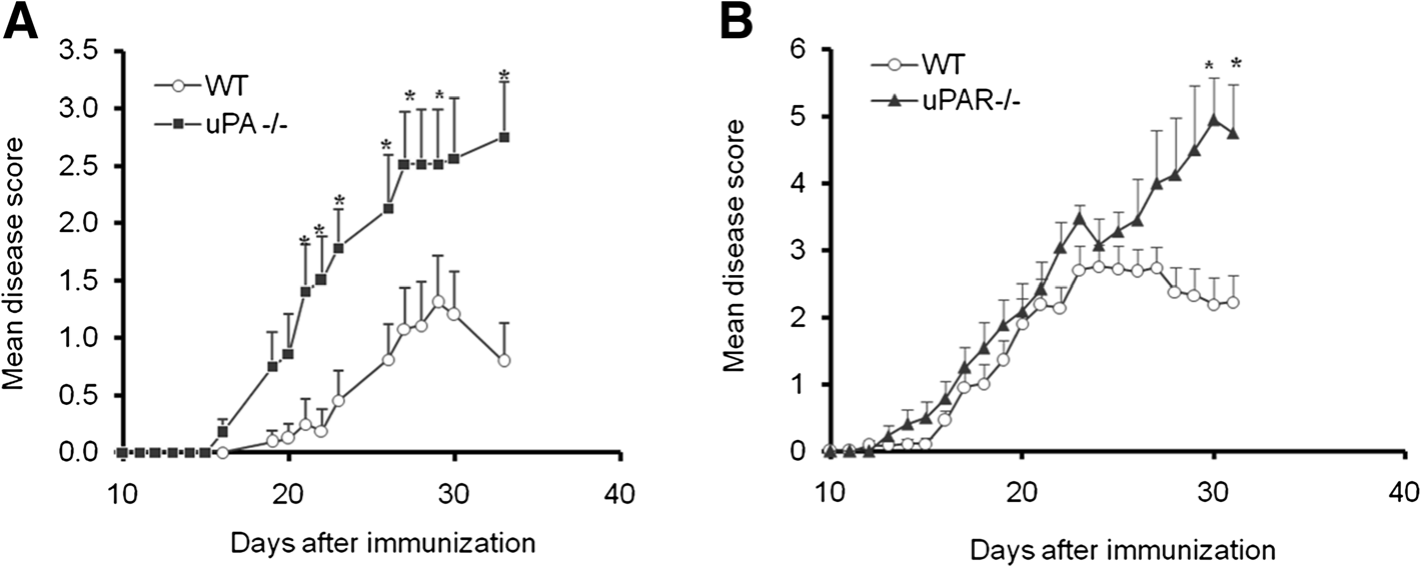
**Experimental autoimmune encephalomyelitis (EAE) is aggravated in mice deficient for the urokinase plasminogen activator (uPA**^**−/−**^**), or the urokinase plasminogen activator receptor (uPAR**^**−/−**^**). (A)** EAE clinical severity in uPA^−/−^ mice and control wild-type (WT) mice. **(B)** EAE clinical severity in uPAR^−/−^ mice and control WT mice. **(A,B)** Results are expressed as the mean clinical score ± standard error (SE) of three separate experiments (*P<0.05).

Several components of the plasminogen activation cascade have been found to be involved in CNS pathologies, including stroke, TBI, MS, and EAE. To evaluate the effect of uPA and uPAR on processes related to CNS inflammation, we examined their involvement in EAE, the experimental inflammatory and demyelinating autoimmune disease used to study human MS. EAE was induced in uPA^−/−^ , uPAR^−/−^, and WT mice. All the KO mice tested presented more severe neurologic scores and delayed recovery compared with the WT mice (Figure 1). In the uPA^−/−^ mice, the mean disease severity was 1.8 ± 0.2 compared with 1.1 ± 0.2 in the WT mice (a 64% increase) (Figure 1A). uPAR^−/−^ mice had significantly more severe disease than the WT mice in the chronic phase of the disease (Figure 1B), with mean disease severity being 2.5 ± 0.4 for uPAR^−/−^ versus 1.7 ± 0.2 for WT (a 47% increase over WT) (Figure 1B). Notably, whereas the WT mice showed remission of the disease about 30 days post-induction, both the uPA^−/−^ and the uPAR^−/−^ animals failed to recover.

### Neuropathologic parameters in uPA^−/−^ and uPAR^−/−^ mice

**Figure 2:**
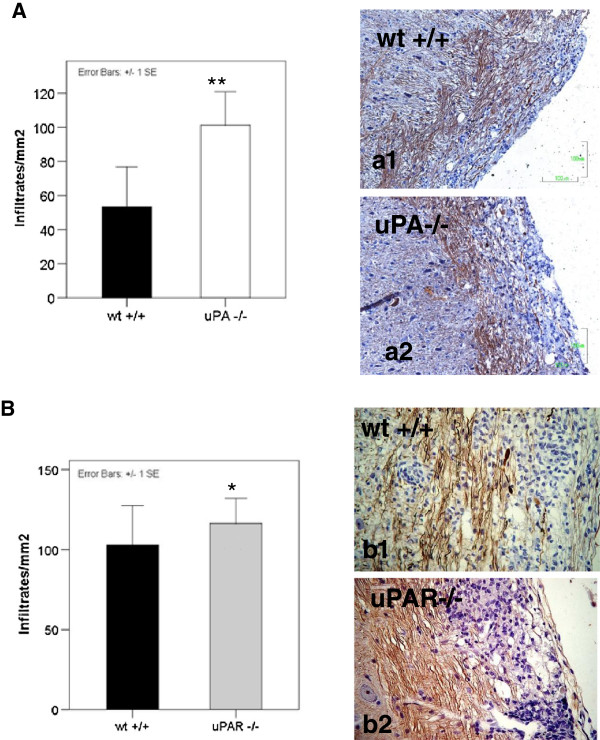
**Neuropathology in the spinal cords of EAE in urokinase plasminogen activator (uPA**^**−/−**^**), or the urokinase plasminogen activator receptor (uPAR**^**−/−**^**) mice compared with WT mice.** The results are presented as mean ± standard error (SE) (infiltrates/mm^2^). **(A)** uPA^−/−^ animals exhibited an almost twofold greater inflammatory burden (**P<0.001). **(a1,a2)** Representative photographs (hematoxylin plus Bielschowsky stain) Magnification x200. **(B)** uPAR^−/−^ animals exhibited significantly greater inflammation (*P<0.05). **(b1,b2)** Representative photographs, magnification x200.

**Figure 3:**
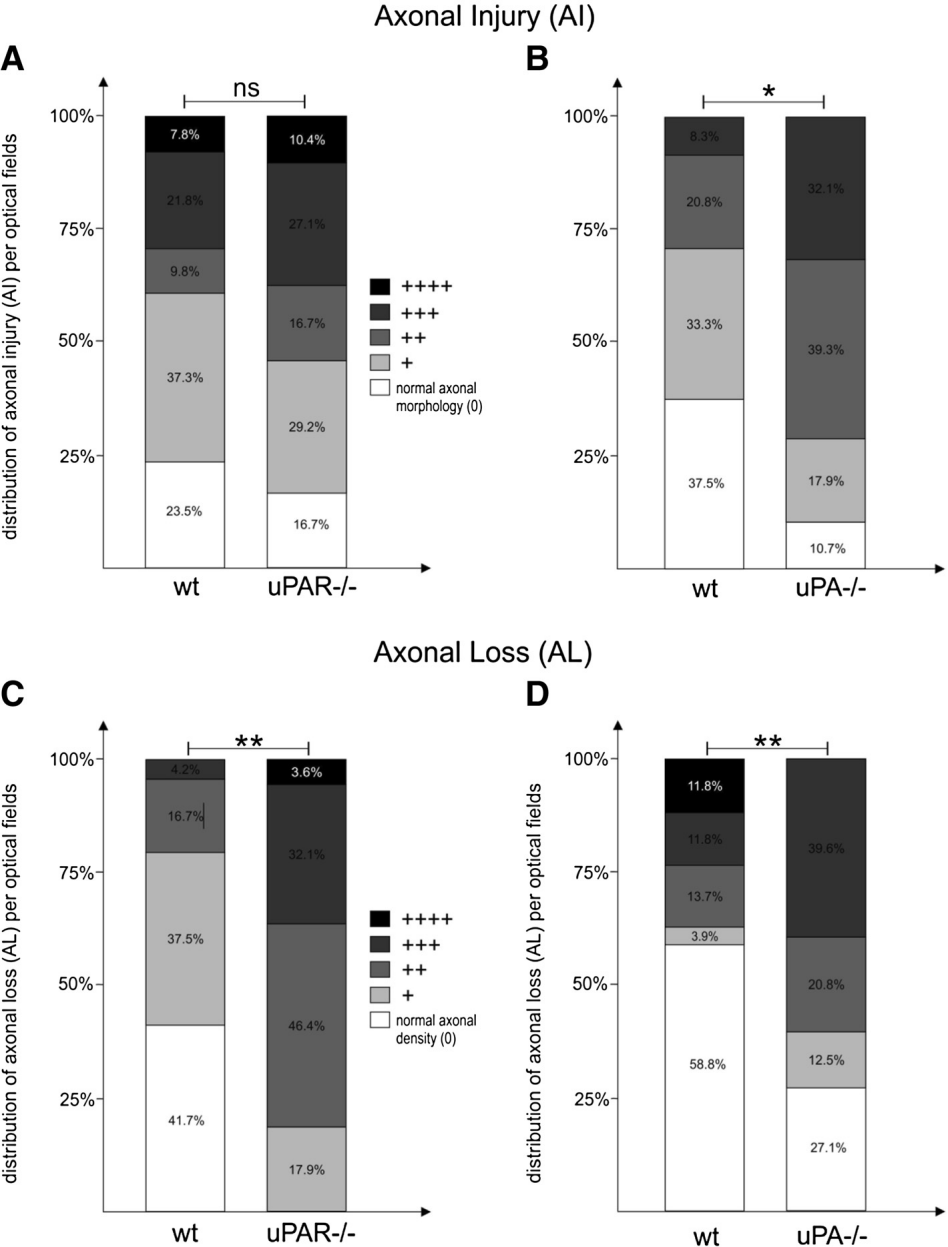
**Axonal injury and axonal loss in experimental autoimmune encephalomyelitis (EAE) in mice deficient for the urokinase plasminogen activator (uPA**^**−/−**^**), or the urokinase plasminogen activator receptor (uPAR**^**−/−**^**) compared with EAE wild-type (WT) mice.** Rsults are presented as percentage distribution of severity. **(A,C)** uPAR^−/−^ mice exhibited **(A)** similarly active axonal injury (AI) and **(C)** significantly greater axonal loss (AL). **(B,D)** uPA^−/−^ mice exhibited **(B)** more severe AI and **(D)** more AL**.** *P<0.05, **P<0.001.

The marked difference in the clinical score of the uPA^−/−^ and uPAR^−/−^ animals was consistent with the neuropathologic processes in their spinal cords during the chronic phase of the disease. Evaluation of cellular infiltration (infiltrates/mm^2^), AI, and AL identified a massive infiltration of mononuclear cells in the spinal cords of the uPA^−/−^ animals (Figure 2A) accompanied by a marked increase in AI and AL (Figure 3B and D) compared with WT EAE mice (*P*<0.001, *p*<0.05, *p*<0.001 respectively). Concomitantly, the uPA^−/−^ group exhibited severe AI with more ovoids, spheroids, and dystrophic axons, even in areas without striking perivascular infiltration, but displayed only some parenchymal infiltrates compared with the corresponding WT controls (Figure 2a1,a2). Milder differences were noted in the brainstem, cerebelli, and hemispheres of the animals (data not shown).

Similarly, the uPAR^−/−^ mice exhibited an increased inflammatory burden in their spinal cords (Figure 2B) (p<0.05) and a marked AL (Figure 3C) (*P*<0.001), albeit with similar levels of AI (Figure 3A) during the chronic phase, compared with the WT EAE-induced mice. These neuropathologic data are in agreement with the clinical courses of the KO and WT animals, which displayed a similar acute phase and a different chronic phase.

**Figure 4:**
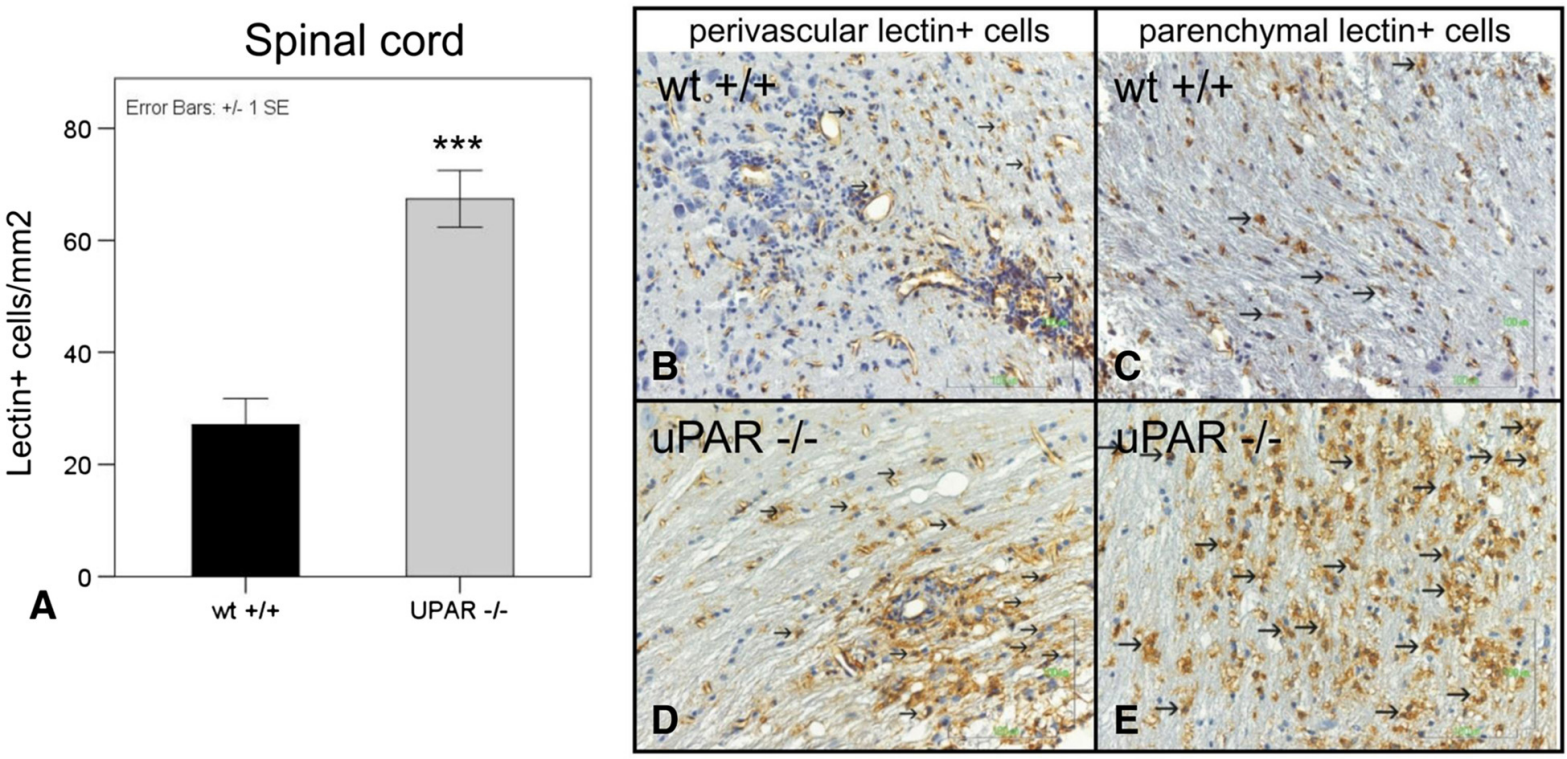
**Lectin-positive microglia/macrophages in spinal cords of mice deficient for the urokinase plasminogen activator receptor (uPAR**^**−/−**^**) and of wild-type (WT) mice. (A)** Significant increase in numbers of lectin-positive cells (microglia/macrophages) in the spinal cords of uPAR^−/−^ animals compared with the WT mice (***P<0.0001). Representative photographs showing perivascular and parenchymal lectin-positive microglia/macrophages (arrows) in **(B,C)** WT and **(D,E)** uPAR^−/−^ animals.

Microglia and blood-borne macrophages play an important role in EAE pathogenesis. Evaluation of activated lectin-positive microglia/macrophages per mm^2^ during the chronic phase of the disease showed a twofold increase within the spinal cords of the uPAR^−/−^ mice compared with the WT control mice (Figure 4).

### Immune responses to MOG_35-55_ and antigen presentation in uPA^−/−^ and uPAR^−/−^ mice

In all the described models of EAE, the cells that initiate the disease are predominantly myelin-reactive CD4^+^ T helper cells. These antigen-specific autoimmune T cells first contact a naive intact BBB, and are able to extravsate through the BBB, because of their active status. In the CNS, as a result of presentation of appropriate antigens, the cells undergo further activation.

**Figure 5:**
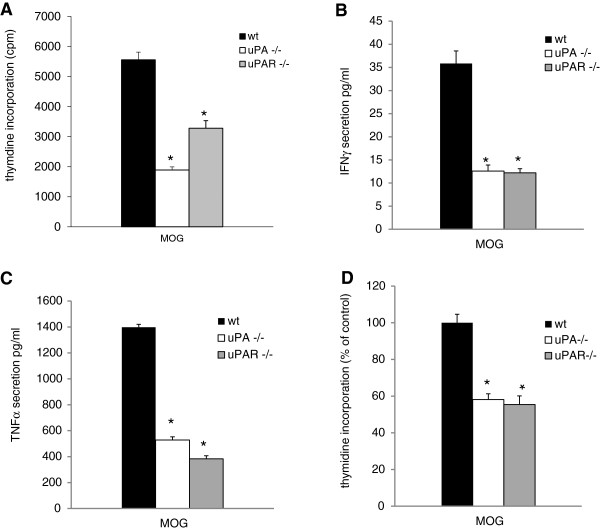
**mice deficient for the urokinase plasminogen activator (uPA**^**−/−**^**), or the urokinase plasminogen activator receptor (uPAR**^**−/−**^**) show reduced T-cell reactivity to myelin oligodendrocyte glycoprotein (MOG)**_**35–55 **_**and reduced antigen presentation. (A**) Proliferation of lymphocyte from all the tested groups was checked by [^3^H] thymidine incorporation in the presence of MOG_35-55_. (**B).** The effect of uPA and uPAR deficiency on T helper 1 (Th1) cytokine secretion. Lymphocytes from uPA^−/−^, uPAR^−/−^ and WT mice were stimulated with MOG_35-55_, the media were collected after 24 hours, and the secreted **(B)** interferon (IFN)-γ and **(C)** tumor necrosis factor (TNF)-α were measured by ELISA. **(A–C)** Results are expressed as mean ± standard error (SE) of three separate experiments (*P<0.05) **(D)** Effect of uPA and uPAR deficiency on antigen presentation measured by MOG_35-55_ specific T-cell proliferation. Lymphocyte proliferation was checked by [^3^H] thymidine incorporation using APCs from knockout (KO) or WT mice in the presence of MOG_35-55_. Results are expressed as the mean ± SE of four separate experiments (*P<0.05).

To assess the differences between the KO mice and the WT mice, we performed two sets of experiments: T-cell activation, evaluated by T-cell proliferation and pro-inflammatory cytokine secretion, and an antigen-presenting assay. Unexpectedly, and unrelated to disease severity, the T-cell reactivity towards the encephalitogenic MOG_35–55_ peptide of cells derived from the uPA^−/−^ and uPAR^−/−^ mice was found to have a significantly lower response to the tested antigen compared with WT cells. This presented as reduced proliferation of MOG_35–55_ encephalitogenic cells (Figure 5A), and reduced secretion of pro-inflammatory cytokines. Absence of uPA resulted in a 57% reduction in IFN-γ secretion and a 62% reduction in tumor necrosis factor (TNF)-α (Figure 5B,C). Similarly, in the absence of uPAR, a 70% reduction in IFN-γ secretion and a 45% reduction in TNF-α secretion were seen.

We then examined the ability of APCs cultured from uPA^−/−^ and uPAR^−/−^ mice to mount an immunologic reaction compared with APCs from WT animals. APCs were prepared from uPA^−/−^, and uPAR^−/−^, and WT animals (as described in Materials and Methods), and plated with anti–MOG_35-55_-specific lymphocytes in the presence of MOG_35-55_. Figure 5D shows a reduction in lymphocyte proliferation in the presence of APCs from uPA^−/−^ and uPAR^−/−^ animals compared with that in the WT animals. The results indicate a reduction in antigen presentation capacity in the uPA^−/−^ and uPAR^−/−^ animals.

### Amelioration of EAE after PAI-1 administration

Cumulatively, these results suggest that in addition to their effect on the inflammatory system, uPA and uPAR play other protective roles. Indeed, uPA and uPAR are known to initiate and stimulate the fibrinolytic system, suggesting that, in sum, the balances of their effects are neuroprotective. In an attempt to inhibit the deleterious, pro-inflammatory effects of the lack of uPA and uPAR, and to increase their pro-fibrinolytic effect, we injected the animals with the PAI-1-derived 18 amino acid peptide (PAI-dp). This peptide correlates with the docking site of PAI-1 within the uPA protein. Binding of the peptide to uPA prevents binding of the intrinsic PAI-1 to uPA, thereby preventing the inhibitory effect of PAI-1. We previously reported that PAI-dp inhibits the binding of uPA to PAI-1 and in so doing, increases the plasminogen activation ability of uPA, and inhibits uPA-induced signal transduction [14].

**Figure 6:**
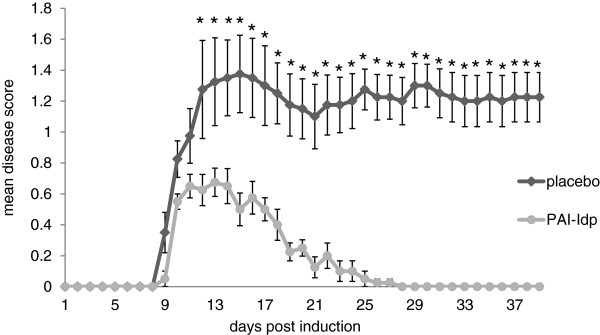
**Administration of the plasminogen activator inhibitor-derived peptide PAI-1dp ameliorated the clinical course of experimental autoimmune encephalomyelitis (EAE).** EAE was induced in wild-tupe (WT) mice by immunization with myelin oligodendrocyte glycoprotein (MOG)_35–55_. The animals were given either placebo or PAI-dp 0.5 mg/kg twice daily by intraperitoneal injection. Results are expressed as the mean clinical score ± standard error (SE) of three separate experiments. (n = 16 for each group). The results are the mean of three separate experiments.

**Figure 7:**
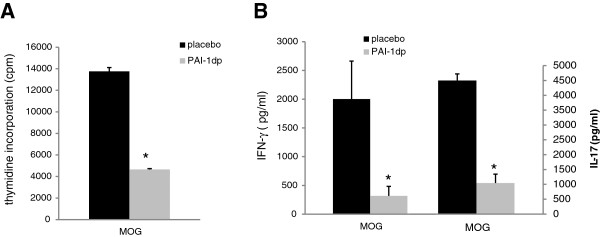
**Administration of PAI-1dp reduces T-cell reactivity towards MOG**_**35-55 **_**peptide. A**. PAI-1dp pre-treatment reduced T cell proliferation induced by MOG_35-55_. **B**. Reduced secretion of the pro-inflammatory cytokines IFN-γ and IL-17. The results are the mean standard error (SE) of three separate experiments (*P<0.05).

WT mice induced with EAE were injected with PAI-1dp, and compared with placebo-injected EAE mice. As seen in Figure 6, preventative administration of PAI-dp markedly and significantly suppressed the disease, with disease severity being reduced by 83% (from 1.2 ± 0.3 to 0.2 ± 0.04). Furthermore, T-cell reactivity towards the encephalitogenic MOG_35–55_ peptide was reduced in the PAI-1dp-injected mice. As shown in Figure 7, T-cell proliferation was reduced (Figure 7A) in lymphocytes derived from PAI-1-injected mice. Accordingly, the pro-inflammatory cytokines IFN-γ and IL-17 were also reduced in these mice (Figure7B).

## Discussion

The data presented in this study show that the PA system plays a role in CNS inflammation. Using mice lacking uPA and uPAR, we found that during EAE, mice exhibit a very severe disease, with impaired recovery. The neuropathologic findings were consistent with disease severity; uPA^−/−^ and uPAR^−/−^ mice showed an increase in AL and a massive infiltration of mononuclear cells into the spinal cord.

In addition, the importance of microglial activation in EAE pathology is known, and we found that microglial activation was increased twofold within the spinal cord of uPAR^−/−^ mice. Microglia are resident APCs that make up to 10 - 20% of all the glial cells in the CNS [19]. In MS and EAE, microglia are activated, express high levels of major histocompatibility complex class II, and function as APCs [20,21]. The increase in lectin-positive microglia/macrophage activation seen in the uPAR^−/−^ animals is in accordance with the clinical severity of the disease.

Our results are in agreement with previously reported results. East *et al*. reported a more severe EAE in tPA^−/−^ mice, characterized by incomplete recovery and increased neurologic deficit, and also reported persisting inflammatory cuffs of mononuclear cells and a greater degree of demyelination in uPAR^−/−^ mice [9]. Another report by East *et al*. showed that mice lacking the plasminogen inhibitor PAI-1 developed milder EAE without clinical relapse, and with an overall reduction in neuroinflammation [22].

These findings, together with those from the earlier part of the current study, prompted us to attempt to treat EAE using the PAI-dp, the peptide derived from the PAI-1 protein. PAI-1dp inhibits the action of PAI-1, thereby prolonging the presence and proteolytic activity of uPA. Importantly, we found that when EAE mice were pre-treated with PAI-1dp, they developed a markedly less severe disease, accompanied by a reduction in T-cell reactivity. To our knowledge, this amelioration of EAE after the novel approach of injecting peptides derived from the PA system has not been reported previously.

The roles played by the PA system in the pathogenesis of EAE could be ascribed to a number of different mechanisms, such as regulation of fibrin deposition at sites of inflammation, and effects on cell trafficking into the CNS. Fibrin, the end product of PA proteolytic activity, participates in a variety of cellular responses associated with inflammation [23,24] by binding to a range of receptors that are expressed on leucocytes, macrophages, and monocytes [25]. Fibrinogen-deficient mice show delayed inflammatory responses to lipopolysaccharide [26]. Because fibrin is capable of modulating inflammation via a number of different mechanisms, it is clear that removal of fibrin by enhancing fibrinolysis can produce beneficial results.

Although the uPA^−/−^ and uPAR^−/−^ mice exhibited more severe disease, there was a reduction in their immune response compared with WT mice. This was reflected by a reduction in proliferative lymphocytes and a reduced ability to present antigen. These findings point to a paradoxical situation in which the KO mice present with more severe disease, a condition that is usually characterized by high lymphocyte proliferation and cytokine induction, but in this study showed just the opposite results. The PA system is considered to be both pro-inflammatory and anti-inflammatory. The former is related to the mounting of correct innate and adaptive immune responses, whereas the latter is related to prevention of extracellular fibrin deposition [13]. The PA system components involvement in two opposing processes might explain these conflicting results.

There is an intriguing link between the uPA/uPAR system and the immune system. Both uPA and uPAR expression were shown to be modulated by inflammatory mediators. TNF-α, IFN-γ, and IL-1 were found to increase uPA expression on macrophages [27,28]. Activated T lymphocytes express uPA and uPAR [29,30], and stimulation with phorbol esters and T-cell receptor (TCR)-mediated stimulation results in substantial upregulation of uPA and uPAR in T cells [31]. uPAR is co-expressed with CD25 (IL-2R), and its expression in TCR-mediated T-cell activation has been established [32]. Furthermore, uPAR is upregulated by exposure to IL-2 and IL-4, but not to several other cytokines [33]. Conversely, there is substantial evidence suggesting that uPA is a modulator of immune and inflammatory responses. For example, early reports showed that PA could act as a lymphocyte mitogen [32]. Thus, bidirectional communication links the uPA/uPAR system and the inflammatory cytokine networks. Therefore, the profound effect of uPA/uPAR deficiency on lymphocyte activation in our model is not unexpected, and the severe clinical outcome can be explained by the inability of the KO mice to mount a protective immune response. Similar results were reported by Gyetko *et al*. [34] who investigated the ability of uPA^−/−^ mice to mount a protective host defense during infection with the opportunistic yeast *Cryptococcus neoformans.* In the absence of uPA, the mice failed to mount an adequate immune defense against the yeast, resulting in a lethal defect in cell-mediated immune responses.

When targeting the PA system for treatment of EAE/MS, it is important to bear in mind its major role in the fibrinolytic system, and that manipulation of the PA system might cause unwanted effects in the coagulation/fibrinolytic system. Recombinant tPA has been in clinical use for treatment of myocardial infarct. However, it can be given only once in such cases, and not as an ongoing treatment because it causes excessive bleeding, and therefore cannot be used for treatment of MS.

It is interesting that mice lacking uPA do not have major thrombotic disorders [35]. This is most probably because of the redundant fibrinolytic function of uPA, which, at least in the vascular compartment, can be substituted for by tPA. Double uPA-tPA KO mice show extensive thrombotic disorders similar to those in plasminogen KO mice [36]. In addition, disruption of the *PAI-I* gene in mice does not appear to impair hemostasis, but is associated with increased resistance to thrombosis and with a mild hyperfibrinolytic state characterized by enhanced *in vivo* clot lysis [37]. Interestingly, despite the minor influence of the disruption of these genes in the naïve state, the effects of disruption become apparent upon disease induction. This suggests that the biologic consequences of PA system gene inactivation might be more significant in disease states.

## Conclusions

It is probable that the PA system plays both harmful and beneficial roles in MS/EAE, and that there is a balance between injury and recovery. This balance may be regulated via the expression and secretion of cytokines and proteolytic enzymes. Our results suggest that attenuation of uPA activity by pre-treatment with PAI-1-dp may be a potential mechanism for treatment of CNS inflammatory and demyelinating diseases.

## Abbreviations

APC: Antigen-presenting cell; BBB: Blood–brain barrier; CFA: complete Freund’s adjuvant; CNS: Central nervous system; EAE: Experimental autoimmune encephalomyelitis; FCS: fetal calf serum; IFN: Interferon; IL: Interleukin; KO: Knockout; LNC: lymph-node cell; MOG: Myelin oligodendrocyte glycoprotein; MMP: Matrix metalloproteinase; MS: Multiple sclerosis; NIH: National Institutes of Health; PA: Plasminogen activator; PAI-1: Plasminogen activator inhibitor protein; PBS: phosphate-buffered saline; TNF: Tumor necrosis factor; tPA: Tissue plasminogen activator; uPA: urokinase plasminogen activator; uPAR: urokinase plasminogen activator receptor; WT: Wild-type.

## Competing interests

The authors declare that they have no competing interest.

## Authors’ contributions

TM, FYMP and DGW performed the animal and cell culture studies. AL and NG performed the pathological studies, the tissue staining and evaluation. TB and AAH were involved in study design. TB and DGW were involved in writing the manuscript. All authors approved the final manuscript.
